# Implementation of a Web-Based Chatbot to Guide Hospital Employees in Returning to Work During the COVID-19 Pandemic: Development and Before-and-After Evaluation

**DOI:** 10.2196/43119

**Published:** 2024-07-25

**Authors:** Ozan Unlu, Aaron Pikcilingis, Jonathan Letourneau, Adam Landman, Rajesh Patel, Erica S Shenoy, Dean Hashimoto, Marvel Kim, Johnny Pellecer, Haipeng Zhang

**Affiliations:** 1 Division of Cardiovascular Medicine Brigham and Women's Hospital Boston, MA United States; 2 Mass General Brigham Boston, MA United States; 3 Harvard Medical School Boston, MA United States; 4 Brigham Digital Innovation Hub Brigham and Women's Hospital Boston, MA United States; 5 Division of Infectious Diseases Massachusetts General Hospital Boston, MA United States; 6 Occupational Health Services Mass General Brigham Boston, MA United States

**Keywords:** chatbot, return to work, employee, health care personnel, COVID-19, conversational agent, occupational health, support service, health care delivery, agile methodology, digital intervention, digital support, work policy, hospital staff

## Abstract

**Background:**

Throughout the COVID-19 pandemic, multiple policies and guidelines were issued and updated for health care personnel (HCP) for COVID-19 testing and returning to work after reporting symptoms, exposures, or infection. The high frequency of changes and complexity of the policies made it difficult for HCP to understand when they needed testing and were eligible to return to work (RTW), which increased calls to Occupational Health Services (OHS), creating a need for other tools to guide HCP. Chatbots have been used as novel tools to facilitate immediate responses to patients’ and employees’ queries about COVID-19, assess symptoms, and guide individuals to appropriate care resources.

**Objective:**

This study aims to describe the development of an RTW chatbot and report its impact on demand for OHS support services during the first Omicron variant surge.

**Methods:**

This study was conducted at Mass General Brigham, an integrated health care system with over 80,000 employees. The RTW chatbot was developed using an agile design methodology. We mapped the RTW policy into a unified flow diagram that included all required questions and recommendations, then built and tested the chatbot using the Microsoft Azure Healthbot Framework. Using chatbot data and OHS call data from December 10, 2021, to February 17, 2022, we compared OHS resource use before and after the deployment of the RTW chatbot, including the number of calls to the OHS hotline, wait times, call length, and time OHS hotline staff spent on the phone. We also assessed Centers for Disease Control and Prevention data for COVID-19 case trends during the study period.

**Results:**

In the 5 weeks post deployment, 5575 users used the RTW chatbot with a mean interaction time of 1 minute and 17 seconds. The highest engagement was on January 25, 2022, with 368 users, which was 2 weeks after the peak of the first Omicron surge in Massachusetts. Among users who completed all the chatbot questions, 461 (71.6%) met the RTW criteria. During the 10 weeks, the median (IQR) number of daily calls that OHS received before and after deployment of the chatbot were 633 (251-934) and 115 (62-167), respectively (*U*=163; *P*<.001). The median time from dialing the OHS phone number to hanging up decreased from 28 minutes and 22 seconds (IQR 25:14-31:05) to 6 minutes and 25 seconds (IQR 5:32-7:08) after chatbot deployment (*U*=169; *P*<.001). Over the 10 weeks, the median time OHS hotline staff spent on the phone declined from 3 hours and 11 minutes (IQR 2:32-4:15) per day to 47 (IQR 42-54) minutes (*U*=193; *P*<.001), saving approximately 16.8 hours per OHS staff member per week.

**Conclusions:**

Using the agile methodology, a chatbot can be rapidly designed and deployed for employees to efficiently receive guidance regarding RTW that complies with the complex and shifting RTW policies, which may reduce use of OHS resources.

## Introduction

To reduce nosocomial COVID-19 infections, on March 16, 2020, consistent with recommendations from the Centers for Disease Control and Prevention (CDC) [1], the Massachusetts Department of Public Health and the Commissioner of Public Health issued an order that all Massachusetts hospitals must screen health care personnel (HCP), patients, and visitors for symptoms of a respiratory infection, and individuals with any such symptoms should not be permitted on site [2]. The order and guidelines prompted health care facilities to develop policies for HCP COVID-19 testing and return to work (RTW) eligibility criteria after reporting symptoms, exposures, or COVID-19 infection. To complicate the landscape further, each US state issued travel advisories for residents and travelers with different requirements for COVID-19 testing and isolation [3]. As the pandemic progressed, ongoing changes to these orders, guidelines, and advisories required health care facilities to make frequent updates to their policies. The high frequency of changes and complexity of the policies made it difficult for HCP to understand when they needed testing and were eligible to RTW. Ongoing staffing shortages, pervasive throughout health care and other industries, elevated the importance of ensuring effective communication to preserve workforce capacity and support workforce wellness [4]. There was an opportunity and need for novel tools to help HCP navigate the latest COVID-19 testing and RTW policies.

Throughout the COVID-19 pandemic, health care facilities sought innovative ways to manage the surging demand for medical information, guidance, and care. One of the substantial technological advancements embraced during this time was the use of chatbots that use natural language to interact with users through text or voice. These digital tools were used to facilitate immediate responses to patients’ and employees’ queries about COVID-19, assess symptoms, and guide individuals to appropriate care resources [5-8].

At Mass General Brigham (MGB), a large integrated health system, Occupational Health Services (OHS) was tasked with implementing and maintaining the RTW policy and other employee health policies about COVID-19. Given the evolving nature of the COVID-19 response, OHS created an MGB COVID-19 OHS hotline as one of the primary ways for employees to get guidance on COVID-19 policies. Rapidly changing and difficult to follow policies led to an increased number of calls to the hospital system’s OHS, drawing resources away from their core functions. During the periods of increased COVID-19 exposure and infection among HCP, the MGB COVID-19 OHS hotline was sometimes overwhelmed with calls from MGB employees, leading to long wait times and frustration among employees and OHS hotline staff. Despite ongoing employee education efforts, the need to support HCP with RTW policies and procedures persisted, prompting the need for novel self-service support resources. The RTW chatbot was designed to interact with HCP to help them navigate the MGB RTW policies, receive specific RTW decision support through a web-based conversational interface, and offload some of the demand from the MGB COVID-19 Hotline. Here, we describe the development of an RTW chatbot and examine the chatbot’s impact on the number and duration of calls received by the OHS hotline.

## Methods

### Setting

This study was conducted at MGB, a not-for-profit, academic, integrated health care delivery system in Massachusetts and New Hampshire with two academic medical centers (Brigham and Women’s Hospital and Massachusetts General Hospital), multiple community and specialty hospitals, a large affiliated physician network, community health centers, a home care organization, a health insurance plan, and other health-related services. MGB is the largest private employer in Massachusetts and has over 80,000 employees. MGB OHS established a system-wide hotline staffed by registered nurses to support queries from employees, referred to herein as HCP. The OHS hotline handled requests for COVID-19 testing and provided guidance regarding RTW requirements, which differed based on whether the HCP was reporting symptoms before COVID-19 diagnosis, had been diagnosed with COVID-19, or had an identified exposure to SARS-CoV-2, consistent with public health guidelines. Additional layers of complexity included changes in time- and test-based RTW criteria, differential management of results from antigen and molecular tests, and differential management based on the immunocompromised status of the HCP.

### Development of the Chatbot

**Figure 1:**
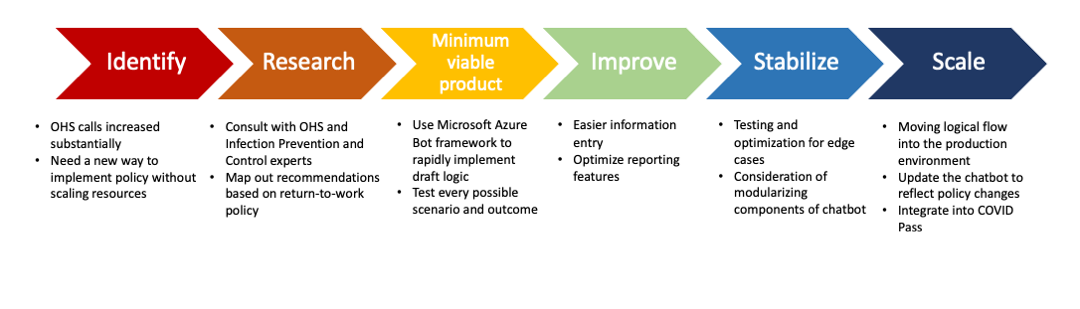
Agile design methodology used in the study. OHS: Occupational Health Services.

During the pandemic, many COVID-19–related technology innovations used agile design methodologies to rapidly deploy solutions in an iterative and efficient manner [5]. Agile methodology is an iterative approach to project management and software development where teams deliver work in small, but consumable, increments. Requirements, plans, and results are evaluated continuously so teams have a natural mechanism for responding to change quickly. We developed the RTW chatbot similarly using an agile design methodology (Figure 1). We worked with leaders from OHS to understand the reasons for the large number of OHS calls at the end of December 2021 and rapidly iterated on potential approaches to address this issue. The RTW chatbot was suggested as a potential approach. We were able to achieve rapid consensus from OHS and Infection Control leadership, and the first iteration of the RTW chatbot was developed within a week.

To create the RTW chatbot, we started by reviewing the existing RTW policies in conjunction with MGB OHS and Infection Control, who jointly developed and maintained the policy. We subsequently mapped the policy into a unified flow diagram that included all required questions and recommendations that an RTW chatbot might provide (Figure S1 in Multimedia Appendix 1).

Once the end points, logic, and recommendations were finalized, we used the Microsoft Azure Health Bot framework to rapidly implement draft logic for further testing and review. Azure Health Bot service is a low-code cloud platform that empowers developers in health care organizations to build and deploy secure, compliant, conversational health care experiences at scale. We used the Azure Bot Framework but turned off the Healthbot features, as they were not relevant to the design of our chatbot. With the logic flow established, we used the Microsoft Azure Health Bot scenario editor to construct a conversational experience that navigates users through the questions to ascertain exposure concerns, symptoms, test results, and the timing of each. The RTW chatbot was designed to calculate when a user could RTW based on user-inputted answers about the onset of symptoms, COVID-19 diagnostic test status and results, and the associated dates for the above, among other user-submitted data points. The RTW chatbot would then tell the HCP whether they were eligible to RTW and provide information on the appropriate next steps the HCP should take if otherwise not cleared to return to on-site work. Importantly, the chatbot was entirely rules based (derived from MGB RTW policy) without any artificial intelligence or machine learning functionality.

**Figure 2:**
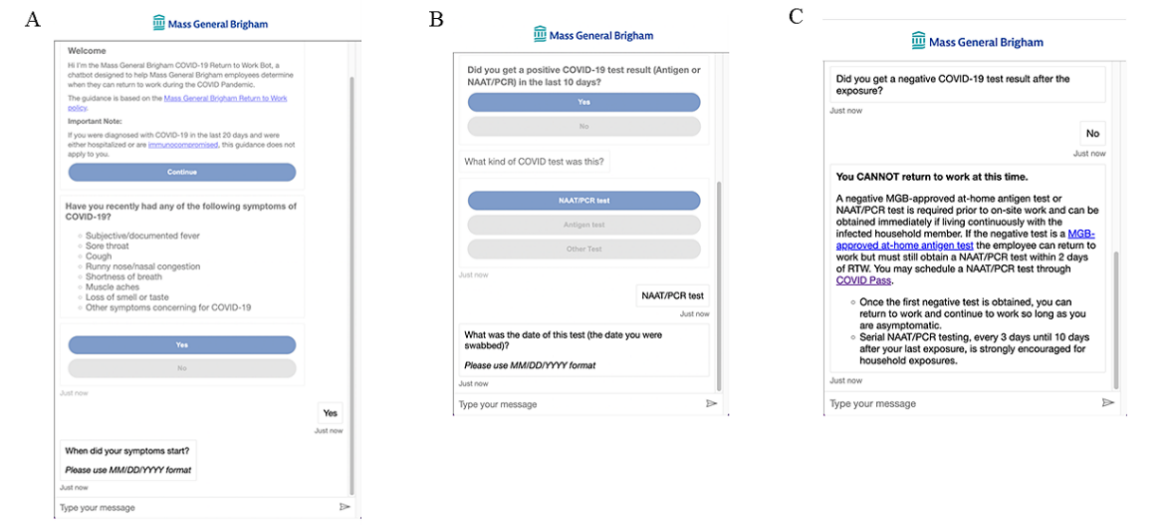
Return-to-work chatbot screenshots: (A) questions related to symptoms, (B) questions related to testing, and (C) "cannot return to work" message.

The project team developed a rigorous quality assurance testing process to test every possible scenario and ensure the RTW chatbot provided accurate recommendations that reflected current RTW policy. For example, if an employee was exposed to a family member who tested positive for COVID-19 within the past 10 days but did not have a negative MGB-approved at-home antigen test, nucleic acid amplification test, or polymerase chain reaction test as required by the policy, the employee was not allowed to RTW (Figure 2). If an employee was deemed appropriate to RTW and followed all steps provided by the RTW chatbot, such as uploading an approved test result, the employee could RTW without calling the OHS Hotline.

We launched the RTW chatbot by moving our logic flow from our development space into a production environment accessible to all HCP on the internal hospital network via a web link. We then updated the OHS RTW intranet web page and all other associated materials, including MGB’s employee daily web-based COVID-19 attestation system, COVID Pass [6], to encourage HCP seeking RTW guidance to use the RTW chatbot. Employees were also notified via email about the availability of the new RTW chatbot, and managers were instructed to direct employees to use the RTW chatbot. These notifications were repeated frequently via various communication channels. In addition, the RTW chatbot was subsequently integrated into COVID Pass [6], the MGB HCP daily symptom attestation tool used throughout the organization.

### Data Collection and Statistical Analysis

The study period was from December 10, 2021, to February 17, 2022, including 5 weeks before and 5 weeks after the deployment of the RTW chatbot. The study period was selected to include 10 weeks with the highest COVID-19 activity and the maximal use of RTW resources. There was also a brief interruption of data collection from the RTW chatbot after 5 weeks of deployment due to a planned update; therefore, we chose to analyze the period with uninterrupted data. We obtained data on the RTW chatbot from Microsoft Azure Bot Framework (Microsoft Corporation). Deidentified OHS call center use data were obtained from pre-existing Tableau dashboards that included details on the use of OHS resources. In addition, we obtained daily COVID-19 cases in Massachusetts reported to the CDC from the publicly available CDC COVID-19 Data Tracker [9].

We performed descriptive statistics on the call volumes of the OHS hotline and the RTW chatbot users. We compared the number of calls, wait times, call times, and abandonment rates of the OHS hotline before and after the introduction of the chatbot. We also calculated the mean time each employee spent to complete an OHS hotline call and to complete the chatbot questions. In addition, we compared the median time spent by each OHS hotline staff before and after the deployment of the RTW chatbot. Finally, we compared the median number of COVID-19 cases in Massachusetts reported to the CDC before and after the deployment of the RTW chatbot.

We calculated means and SDs for continuous variables that are normally distributed and medians and IQRs for continuous variables that are not normally distributed. We compared the number of calls, wait times, call times, and abandonment rates of the OHS hotline using the Mann-Whitney *U* test. Statistical analyses were performed using Python version 3.10.6 (Python Software Foundation).

### Ethical Considerations

The MGB Human Research Office reviewed the study protocol and determined that activities described in this protocol do not constitute human subjects research and deemed it exempt from an institutional review board approval. The data sets used in this study did not include any individually identifiable health information.

## Results

### OHS Call Volume and RTW Chatbot Use

**Figure 3:**
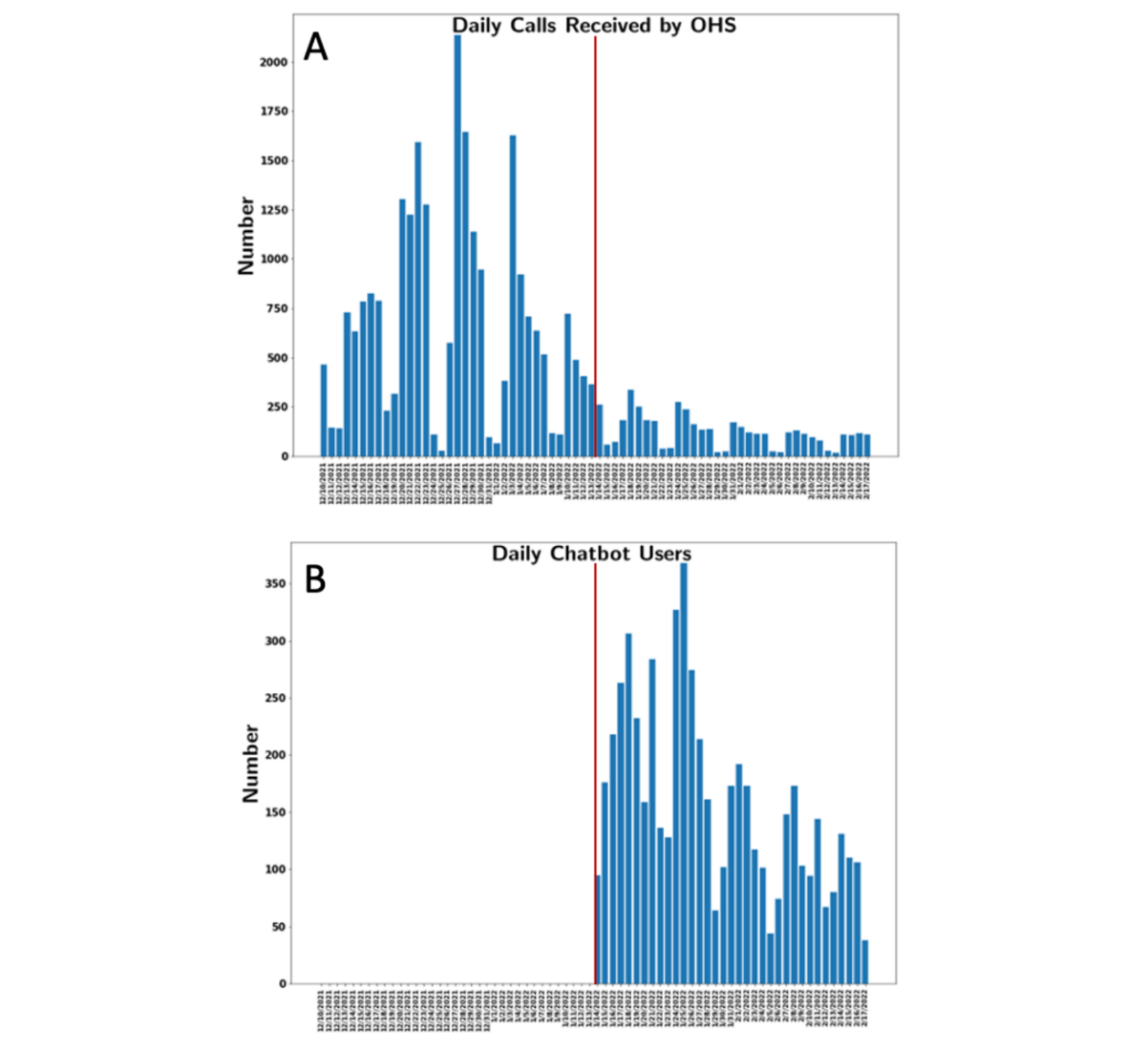
Daily calls received by the occupational health services and daily chatbot users over the study period. OHS: Occupational Health Services.

In the first 5 weeks of the study period, before the RTW chatbot was deployed, the OHS call center received 24,212 calls. In the 5 weeks following the deployment of the RTW chatbot, the number of calls that OHS received decreased from 2726 to 1351 calls from the preceding week to 1 week after the deployment of the RTW chatbot. During the total 10 weeks, the median number of daily calls that OHS received before and after deployment of the chatbot were 633 (IQR 251-934) and 115 (IQR 62-167), respectively (*U*=163; *P*<.001; Figure 3).

The RTW chatbot was only used 98 times on the first day. However, use rapidly increased, and within 1 week of deployment, the RTW chatbot was used by a total of 1449 users. The highest engagement was around January 25, 2022, with 368 users, which was 2 weeks after the peak of COVID-19 in Massachusetts. Lower levels of RTW chatbot activity were observed on weekends. Corresponding with the decline in reported COVID-19 cases within Massachusetts, the chatbot’s daily engagement declined, down to an average of approximately 100 users by the end of the 5-week period. Within the 5 weeks post deployment, a total of 5575 users used the RTW chatbot, and 643 (11.5%) completed all the questions, which was potentially underestimated due to the coding of the final information regarding the appropriateness of returning to work as a prompt step rather than a final step. Among users who completed all the questions, 461 (71.6%) were determined to meet RTW criteria.

### Use Details

Over the entire 10-week study period, the mean OHS call time was 26 minutes and 4 seconds; a mean of 12 minutes and 18 seconds of this was hold time. Throughout the study period, 67.6% (19,285/28,549) of calls were abandoned before being answered. The median OHS call time decreased from 28 minutes and 22 seconds (IQR 25:14-31:05) to 6 minutes and 25 seconds (IQR 5:32-7:08) after the deployment of the chatbot (*U*=169; *P*<.001). Hold time also decreased from 20 minutes and 43 seconds (IQR 18:13-23:52) to 24 (IQR 20-30) seconds (*U*=152; *P*<.001). Similarly, the call abandonment rate decreased from 75.3% to 24.1% (*U*=31; *P*<.001). The median time an OHS hotline staff spent on the phone declined from 3 hours and 11 minutes (IQR 2:32-4:15) per day to 47 (IQR 42-54) minutes per day (*U*=193; *P*<.001) over the 10 weeks, saving approximately 16.8 hours per OHS staff member per week. Notably, the mean time to complete the RTW chatbot was 1 minute and 17 seconds.

### COVID-19 Cases During the Study Period

**Figure 4:**
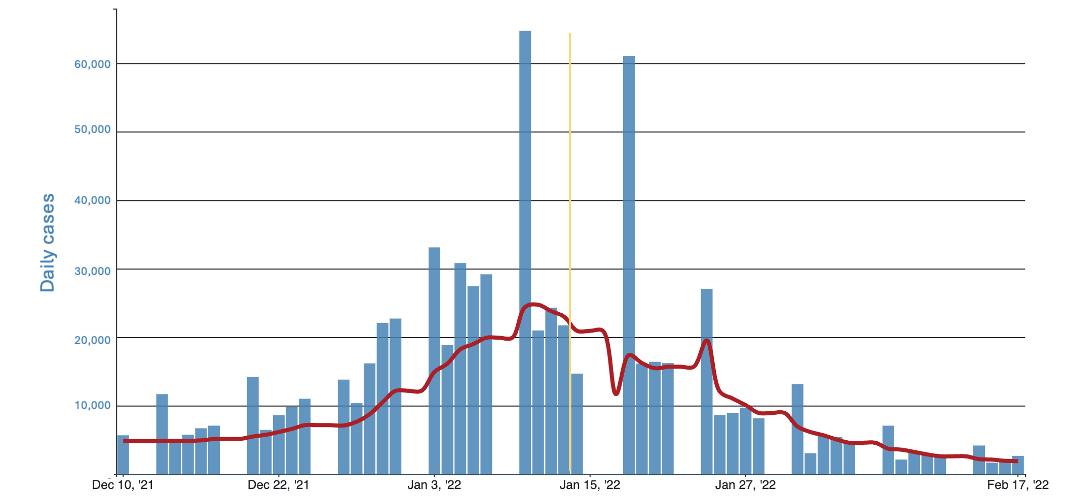
Daily trends in number of COVID-19 cases in Massachusetts reported to the CDC. CDC: Centers for Disease Control and Prevention.

The reported COVID-19 cases in Massachusetts consistently increased during the 5 weeks preceding the deployment of the RTW chatbot and peaked on January 11, 2022, three days before the deployment of the chatbot. The total numbers of reported COVID-19 cases were 388,950 and 316,732 in the 5 weeks preceding and following the deployment of the RTW chatbot, respectively. The median numbers of COVID-19 cases were similar before and after the deployment of the chatbot (7149, IQR 5125-18218 and 6939, IQR 3232-15,671, respectively; *U*=439; *P*=.07; Figure 4).

## Discussion

In this study investigating the development of an RTW chatbot and its impact on OHS call volume and duration, there were several important findings. First, we found that the RTW chatbot was a feasible solution to help HCP navigate a complicated and changing RTW policy. We were able to rapidly design and deploy a web-based tool with consistent HCP use. The dynamic design allowed for ongoing adjustments to chatbot content to align with changing public health recommendations and organizational policy changes. Second, we found that the number of calls that OHS received significantly decreased after the deployment of the RTW chatbot, suggesting the solution’s efficacy. Finally, time spent completing interactions with the RTW chatbot was significantly less than the time spent on calls with OHS, thus it saved both OHS staff and MGB HCP a significant number of hours, reflecting the potential efficiency and cost-saving aspects of this approach.

Among several key challenges in developing and deploying the chatbot were translating policy for edge cases and creating a testing matrix to validate the logic used by the chatbot. The development team worked closely with MGB Infection Prevention and Control and OHS leaders to map all end points to specific recommendations. Unlike the existing tip sheets, the RTW chatbot needed to be “smart” enough to accept and store date inputs and calculate the recommended course of action for each employee. The RTW chatbot needed to parse dates including symptom onset, initial test date, a follow-up test date, and a symptom resolution date. The task of translating policy into logical flows involved taking phrases such as “5 days have elapsed from the positive test (day 0 = day of positive test or symptom onset, whichever is later; return on day 6)” and ensuring the chatbot calculated the correct date, gathered necessary information from employees, and effectively determined if the date entered met the specific criteria.

As the development team created an adaptive decision tree for each use case, an agile, iterative process was key to keeping to a strict timeline. This decision tree for all potential use cases did not exist in a singular material form. To keep the MGB RTW Policy concise, the existing reference documents explained the policy at a high level [10], resulting in “buckets” of HCP outcomes. With the addition of date logic, the RTW chatbot needed extensive quality assurance testing before release. This directed the development team to create a highly detailed matrix of all possible outcomes based on all possible date inputs an HCP might enter. The resulting document took a substantial amount of time and internal resources to compile and test the RTW chatbot.

Although agile processes helped to overcome initial challenges and rapidly deploy the RTW chatbot, rapid development also led to errors in the design, especially for features that would affect the user analytics data collection. In the first iteration of the chatbot, the final information that the HCP received from the chatbot regarding the appropriateness of returning to work was incorrectly coded as a prompt step, which waits for an answer from the user and times-out if there is no input. Though this did not impact the user experience or the accuracy of the recommendations provided by the chatbot, it likely resulted in an underestimation of the true number of users who completed all questions due to an overestimation of the final chatbot prompt time-outs leading to an inflated number of incomplete user journeys through the RTW chatbot.

Chatbots have been used in the COVID-19 pandemic as an effective tool for navigating COVID-19–related policies and information for the public [11]. They have also been used to screen health care employees for COVID-19 symptoms [5]; however, they have not previously been shown to help HCP navigate RTW policies and reduce demand for OHS support. Many states recommend enacting non–health care employee COVID-19 screening tools, which also require policies to manage and support the return of these employees to work after COVID-19 exposures and infections [12]. Chatbot solutions like ours might also help large non–health care employers to enact similar user-friendly and dynamic tools. Looking ahead, similar chatbot tools and the processes we used to create, test, deploy, and iterate could help address similar workplace challenges, especially if we encounter a new pandemic. The RTW chatbot could also be valuable for more routine, nonpandemic conditions, such as when to RTW after influenza infection or gastroenteritis.

There were several limitations in this study. First, it was conducted at a single health system; however, MGB is one of the largest not-for-profit integrated health care delivery systems in the country, representing a variety of health care delivery settings across multiple sites of care. Second, our analyses of the impact on the volume and duration of OHS calls cover a time period during which there were other, parallel efforts, including HCP educational efforts and distribution of MGB-approved at-home antigen tests, which could reduce the number of OHS calls outside of the RTW chatbot implementation. Our study describes an association of the RTW chatbot with reductions in the number and duration of OHS hotline calls but not causation. Finally, the number of reported COVID-19 cases declined after the deployment of the chatbot, which might partially account for the decline in calls OHS received. However, the median number of COVID-19 cases was not significantly different in the 5 weeks preceding and following the deployment of the RTW chatbot, which suggests that the decline in the OHC call volume cannot be solely explained by the decline in cases. In addition, the rate of decline in the OHS call volume was more rapid than the rate of decline in COVID-19 cases in Massachusetts [9,13] or wastewater data [14] (Figure S2 in Multimedia Appendix 1), which suggests a positive impact of MGB RTW guidance efforts, including the RTW chatbot.

Using agile methodology, a chatbot can be rapidly designed and deployed to enable employees to efficiently receive guidance regarding returning to work that complies with complex and shifting RTW policies. We showed that deploying chatbot technology can play a role in decreasing the number and duration of calls OHS receives, reducing the time employees spend determining their ability to RTW and reducing the burden and costs on OHS.

## Appendix

Multimedia Appendix 1 Unified flow diagram including the mapped return-to-work policy and Massachusetts Water Resources Authority's wastewater COVID-19 tracking data.

## Abbreviations

CDC: Centers for Disease Control and Prevention
HCP: health care personnel
MGB: Mass General Brigham
OHS: Occupational Health Services
RTW: return to work

## Data Availability

The occupational health department data sets generated or analyzed during this study are not publicly available given that it is restricted Mass General Brigham occupational health data for employees. The chatbot data analyzed in this study are available from the corresponding author upon reasonable request.
