# Supplementary material for: Implementation of a Web-Based Chatbot to Guide Hospital Employees in Returning to Work During the COVID-19 Pandemic: Development and Before-and-After Evaluation
Source: JMIR Form Res. 2024 Jul 25;8:e43119. doi: 10.2196/43119 (PMC11310642; doi:10.2196/43119)

**Figure S1:** Unified Flow Diagram Including the Mapped Return-to-Work Policy


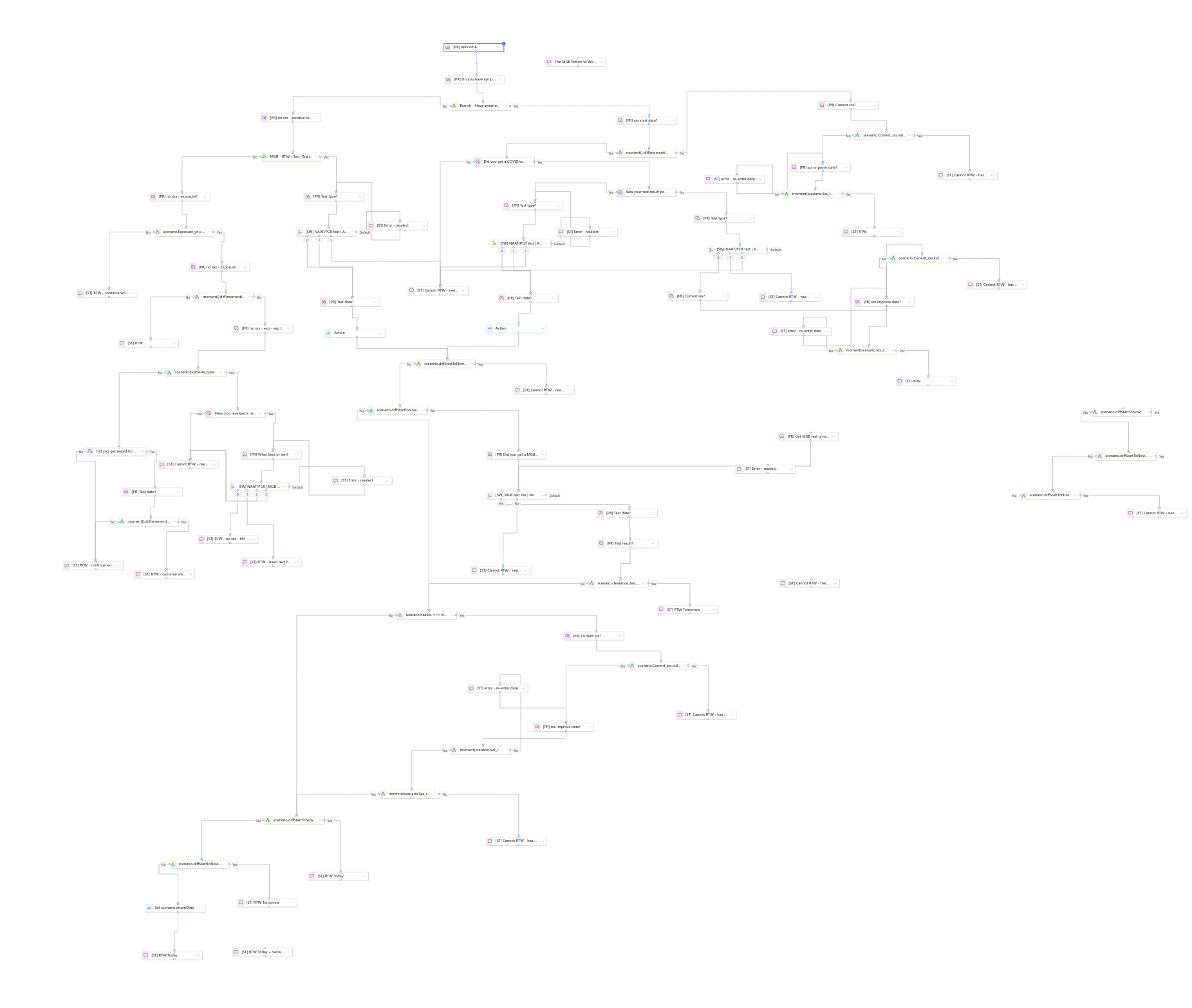


**Figure S2:** Massachusetts Water Resources Authority - Wastewater COVID-19 Tracking Data

**Figure S2 Legend:** The graphs include error bars which represent the range of result variability caused by laboratory processing. The graph with the green dots and error bars represent the North System (B), the graph with the orange dots and error bars represent the South System (C)

A. DITP Viral RNA Signal by Date


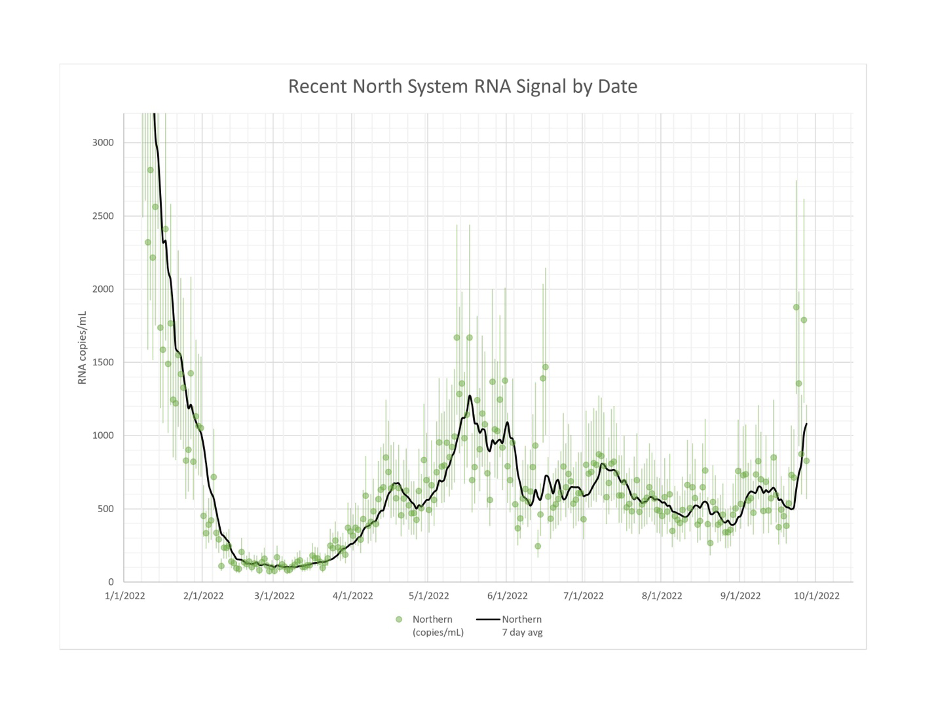


B. Recent North System RNA Signal by Date


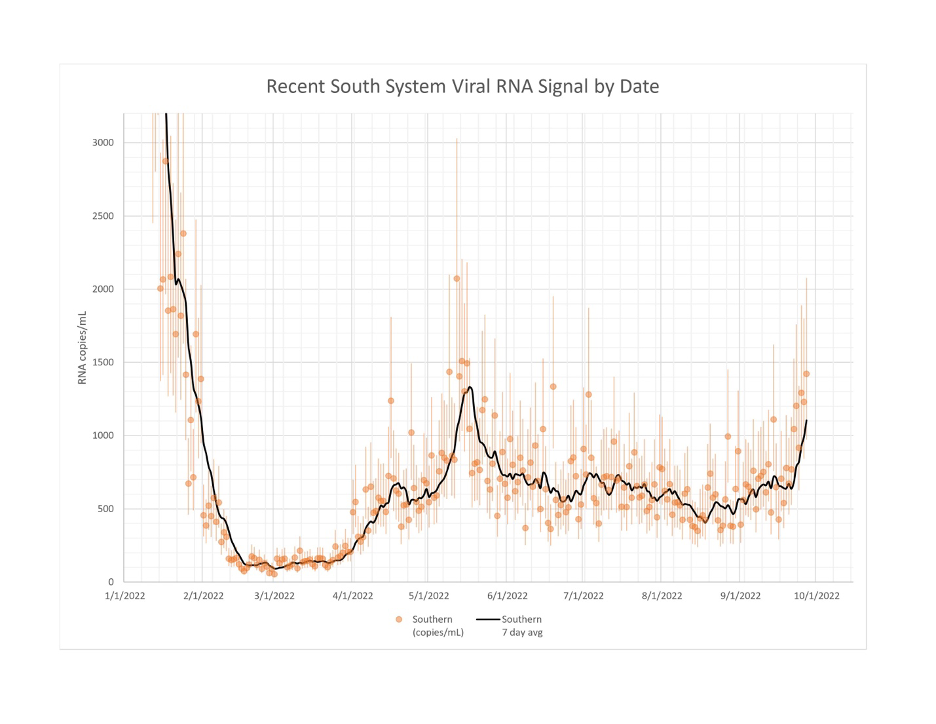


C. Recent South System Viral Signal by Date


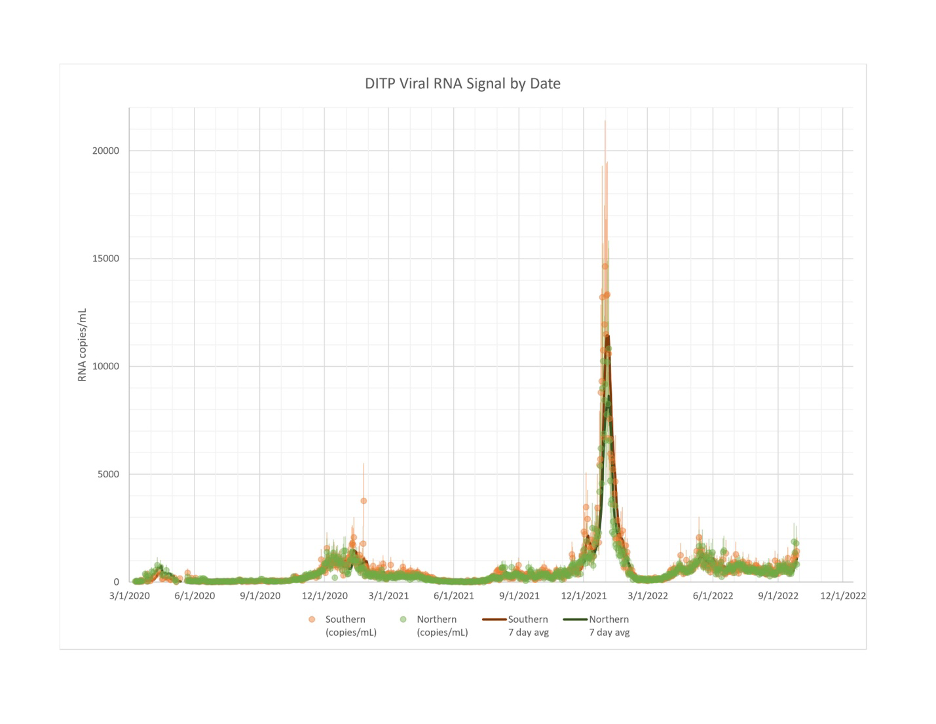

Supplement: Multimedia Appendix 1 [file formative_v8i1e43119_app1.docx]
